# Data for evaluation of the onshore Cretaceous Zululand Basin in South Africa for geological CO_2_ storage

**DOI:** 10.1016/j.dib.2021.107679

**Published:** 2021-12-04

**Authors:** L.V. Tibane, P. Harris, H. Pöllmann, F.L. Ndongani, B. Landman, W. Altermann

**Affiliations:** a Department of Geology, University of Pretoria, Lynwood Road, Pretoria, South Africa; b Mineralogy/Geochemistry, Martin-Luther-University, Halle-Wittenberg, Germany; c Department of Geology, University of Johannesburg, Johannesburg, South Africa; d TerraCore Africa, GeoSpectral Imaging, City of Johannesburg, Gauteng, South Africa

**Keywords:** Sandstones and siltstones lithology, Mineralogy, Geochemistry, Geological CO_2_ sequestration, CO_2_-H_2_O-rock interactions, Geomechanics

## Abstract

The world has set the goal of reducing CO_2_ emissions from burning fossil fuels by using carbon capture and storage (CCS) as one of the major solutions. A sudden and complete switch from fossil fuels to renewable resources cannot be achieved immediately. Therefore, CCS remains an essential techniques to reduce CO_2_. In this work, the 180 – 65 Ma old onshore part of the Zululand Basin in KwaZulu-Natal in South Africa was investigated for geological CO_2_ sequestration. A total of 160 core samples of sandstone, conglomerate, tuff, rhyolite, breccia, and siltstone were taken from NZA, ZA, ZB, and ZC drill cores. The wells were drilled in the 1960s by the South African Petroleum and Gas Corporation Company for hydrocarbon exploration. In order to examine the basin suitability for CO_2_ storage, porosity and permeability, mineralogy, geochemistry, geomechanical properties, and H_2_O-CO_2_-rock interactions were investigated using geological core logging, spectral scanning, petrography, X-ray diffraction (XRD), X-ray fluorescence (XRF), inductively coupled plasma mass spectrometry, uniaxial compressive stress, and scanning electron microscopy. The basin comprises clastic sedimentary rocks, pyroclastic deposits and carbonates from the Makatini, Mzinene and St. Lucia formations. Aptian and Cenomanian sandstones are identified as CO_2_ reservoirs, and the siltstone above is considered capstone. The sandstone comprises on average 34.45 wt% quartz, 32.91 wt% clays, 29.53 wt% feldspars, 4.44 wt% carbonates, 3.10 wt% Fe-oxides, 2.40 wt% micas, and 2.00 wt% organic materials as per XRD data, also contains trace amounts of sulphides and sulphates. Geochemical XRF data for sandstone are 29.72 – 62.51 wt% SiO_2_, 6.95 – 13.44 wt% Al_2_O_3_, 3.06 – 48.81 wt%, 1.90 – 4.51 wt% MgO, 1.04 – 2.19 wt% K_2_O, 1.00 – 3.67 Na_2_O wt%. The content of TiO_2_, Cr_2_O_3_ and P_2_O_5_ is below 0.01 wt% each. Siltstone has similar mineralogy and geochemistry as sandstone, but high clay content, fine-grained, impervious, with porosity <5%. The sandstone and siltstone are geomechanically soft and recorded 15 MPa on the Enerpac P141 device. CO_2_–H_2_O–rock interaction experiments performed at 100 °C and 100 bar using autoclaves showed that sandstone and siltstone react with scCO_2_.

## Specifications Table

**Table:** 

Subject	Carbon capture and storage
Specific subject area	Earth and Planetary Sciences: Geology
Type of data	Tables, images, charts, graphs, and figures
How the data were acquired	The data were obtained through general geological core measurements using log sheets to record lithological changes and geological structures. This method was complemented by using spectral imaging instrument sisuRock Mobile (sisuMOBI) with accurate spectral processing. IntelliCore software was used to discover and identify the exact mineral relationships. Lithological, structural and mineralogy data were collected from the petrographic description of the drill core, supplemented by the use of uncovered thin sections examined under a transmitted light microscope, Olympus CX43 LED, equipped with a universal condenser. In addition, the data were generated using X-ray diffraction (XRD) performed on a Bruker D8 Advance instrument equipped with a 2.2 kW Cu fine focus tube (Cu Kα, λ = 1.54060) and a 90-position sample changer. The geochemical data was acquired through the application of a PANalytical Axios X-ray fluorescence (XRF) spectrometer equipped with a 4 kW Rh tube, performed on fused 1g heated samples and 9.5g flux consisting of 70.69% Li_2_B4O_7_, 19.78% LiBO_2_ and 0.50% LiI at 950 °C. The samples were pressed into a powder briquette by a hydraulic press with the applied pressure of 25 ton. An inductively coupled plasma mass spectrometry (ICP-MS), an Agilent 7500ce quadrupole ICP-MS G3272A equipped with a Babington nebulizer was used for the determination of metal data in residual materials of the dissolved sandstones. The instrument settings (power 1.4 kW, Nebulizer gas flow 0.81 L/min, Plasma flow 18 L/min, spray chamber temperature of 10 °C) were configured according to the manufacturer's specifications.Using transmitted light microscopy and scanning electron microscopy (SEM), JEOL JSM-6300 version, the mineralogical and structural changes as well as porosity and permeability data were recorded. The data were further supported by using a transmitted light microscope to view uncovered thin sections dyed with blue epoxy.
	The geomechanical data was produced by measuring the uniaxial compressive stress by applying force using Enerpac P141 tool. This was performed at an ambient temperature of 25 °C at a single speed, hydraulic hand pump, 327 cm3 and 12.7 mm cylinder stroke, operated at the pressure of 700 bar. The data for sandstone and siltstone reactivity with scCO_2_ was generated by performing H_2_O-CO_2_-rock interactions experiments using autoclaves under 100 °C and 100 bars over a period of four weeks.
Data format	Raw, analysed, processed and filtered
Description of data collection	Ten boreholes have been drilled on the onshore Cretaceous Zululand Basin and are available in the National Core Library of the Council for Geosciences in Pretoria. ZD 1/17, ZE 1/17, ZF 1/72, ZG 1/72, ZH1, and ZU1 are composed of rock and mud fragments that are heavily weathered and altered, and have not been considered in further geochemical and geomechanical investigations [1]. The NZA, ZA, ZB, and ZC cores are relatively well preserved and used to provide geological data on the suitability of the onshore portion of the basin for CO_2_ storage. NZA penetrated to a shallow depth of 571.19 m for the purpose of CO_2_ storage, and no possibility of sandstone reservoir or siltstone was confirmed. Therefore, NZA was considered only for the purpose of lateral borehole correlation.
Data source location	Location of Drill core: The National Core Library at Donkerhoek in Pretoria at S25°47′25.59″, E28°27′34.83″
Data accessibility	Processed data are with the article: DOI:10.1016/j.ijggc.2021.103364 and the raw data are stored in an external depository Mendeley Data and accessible via a link: https://data.mendeley.com/datasets/nzsskyy7dy/1
Related research article	L.V. Tibane, H. Pöllmann, F. Ndongani, B. Landman, W. Altermann, 2021. Evaluation of the lithofacies, petrography, mineralogy, and geochemistry of the onshore Cretaceous Zululand Basin in South Africa for geological CO_2_ storage. International Journal of Greenhouse Gas Control. 109, 103364. DOI:10.1016/j.ijggc.2021.103364.

## Value of the Data

• The data for mineralogy, geochemistry, lateral and vertical variability, porosity and permeability, geomechanical properties, and CO_2_–H_2_O–rock interactions experiments are critical for examining CO_2_ storage potential of the reservoir rock and the caprock of the onshore Zululand Basin.
• The data provide the scientific community in South Africa and other parts of the world with the geological details needed to address the uncertainty regarding the potential CO_2_ sequestration in the Zululand Basin.
• Experimental data on CO_2_–H_2_O–rock interactions from the Zululand basin sandstones and siltstones form the main basis for conducting similar investigations.
• These data can be reused for further insights in developing future scientific research using newly drilled boreholes for the planning, design and development of CO_2_ injection techniques.

## 1 Data Description

### 1.1 Geological core logging

Ten cores were drilled on the onshore Zululand Basin located at S27°42′03″, E32°68′10″. At the time of investigation, the drill cores were stored at the National Core Library (S25°47′25.59″, E28°27′34.83″), managed by the Council for Geoscience. The locations of the individual boreholes are provided in Fig. 1 in [1]. Upon physical inspection, it was discovered that ZD 1/71, ZE 1/17, ZF 1/72, ZH1, ZG 1/72 (Table 1) comprise mud chips and they were not considered for further mineralogical investigation for CO_2_ storage. However, ZA, ZB, ZC, and NZA were well preserved and intact, therefore were washed, logged, sampled and analysed for CO_2_ geological storage [1].

**Fig. 1:**
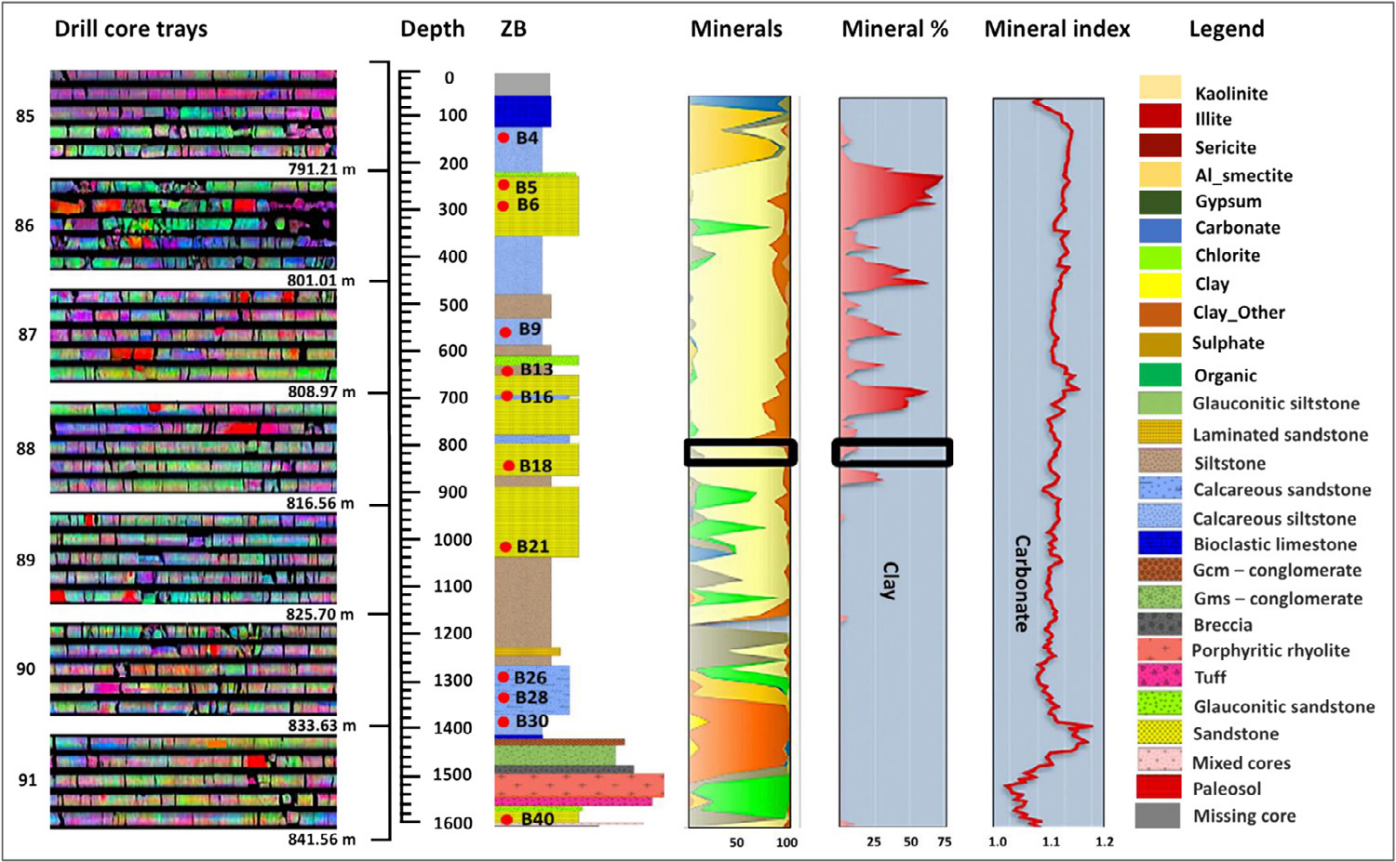
Borehole ZB core trays, core log, mineral distribution, clay mineral percentage, and Fe-carbonate variation with depth, modified from [1]. The mineral column shows that carbonate content is lowest at the depth between 1602 m and 1500 m where volcanic lithofacies occur and highest at 1400 m and 100 m where limestones occur. Clay minerals are dominant at the depth of 850 m.

**Table 1:** Borehole identification, penetration depth in meters, and general description.

Borehole ID	Depth (m)	Description
ZD 1/71	324.61	Core chips exist in 1.5 m intervals. 29.87 m length, exists in 5 boxes between 174.04 m and 558.70 m depth and about half the core consists of intrusive rocks.
ZE 1/17	579.12	These cores were very fragmented, core chips exist in 1.5 m intervals, but lacked intact hard materials which are required for sampling to allow thin section preparations. These cores contained intrusive rocks which make cutting the rocks for thin section preparation difficult and they were considered not suitable for this investigation.
ZF 1/72	527.30	
ZG 1/72	461.77	
ZH1	ca. 500	Comprise core chips, highly weathered and altered [1]. These drill cores were not considered for further investigation.
ZU1	ca. 500	
ZA	1600	Comprised intact cores, packed in 200 core trays. ZB comprised intact cores, packed in 200 core boxes of which box 4 and box 5 at the depth of 98.87 – 100.58 m were missing, probably lost during drilling. ZC comprised intact cores, packed in 220 core boxes and NZA comprised intact cores, packed in 31 core trays. These cores were geologically logged, sampled and investigated further by using X-ray diffraction, X-ray fluorescence, thin sections, scanning electron microscopy, and experimentally reacted with supercritical CO_2_.
ZB	1602	
ZC	2000	
NZA	579.12	

### 1.2 Sample description

According to Tibane et al. [1], the onshore part of the Zululand basin consists of bioclastic limestone, arkosic sandstone, planar laminated sandstone, glauconitic sandstone, calcareous sandstone, massive carbonaceous sandstone, argillaceous siltstone, glauconitic siltstone, calcareous siltstone, clast-supported conglomerate, matrix-supported conglomerate, ash fuff, breccia, and porphyritic rhyolite (Table 2).

**Table 2:** Core logging sheet comprising lithologies, sample identification number and sampling depth intervals in meters, and the general descriptions of 40 core samples collected from borehole ZB.

Lithofacies, sample ID, and depth (m)	Descriptions of Borehole ZB lithologies and samples
Bioclastic limestone B1 (71.93 – 71.58) B2 (73.76 – 73.66) B3 (101.19 – 101.04) B8 (491.64 – 491.49) B10 (604.80 – 604.70) B14 (712.01 – 711.96) B15 (738.53 – 738.38) B20 (988.16 – 988.03) B30 (1406.65 – 1406.45)	The bioclastic limestone samples are grey and white owing to the abundance of calcite formed by the accumulation of skeletal and non-skeletal, elongated and spherical fragments ranging from micrometres to centimetres sizes. Sometimes the limestones occur as thin layers of 20 cm thick interbedded within calcareous sandstone and calcareous siltstone. The bioclastic limestones appear weakly indurated, massive and relatively hard to break with bare hands. Fossil fragments ranging from less than 1 mm to ca. 5 cm length occur. The samples are composed of fine- to medium-grained generally 1.0 mm wide and up to 2.3 mm long bioclasts of echinoderms, algae, bivalves, foraminifera, and gastropods. Lenses of crystallised calcite of 1 mm thickness and 2 to 4 cm long were observable with large bivalve fragments up to 30 mm cemented in blocky calcite. The aforementioned fossils occur embedded together with sub–rounded monocrystalline quartz, calcite, and smectite matrix within calcite cement. The calcite content ranges from 50% to 95% with an average of 75% and occur with10% quartz, 5% plagioclase, 5% clay content and <5% mica. The bioclastic limestones were deposited in shallow marine as indicated by the appearance of shells of organisms and carbonate minerals and supported by sparse glauconite. The samples react with 3% hydrochloric acid owing to the occurrence of carbonate materials and produce whitish effervescence.

Arkosic sandstone B18 (869.39 – 869.29) B21 (1046.99 – 1046.81) B22 (1093.32 – 1093.27) B23 (1126.39 – 1126.29) B29 (1214.63 – 1214.48) B35 (1470.36 – 1462.74) B40 (1570.12 – 1570.02) Argillaceous siltstone B9 (551.38 – 551.28) B19 (924.86 – 924.74)	The arkosic sandstone and argillaceous siltstones are greyish, moderately indurated, compacted and massive, they are relatively hard to break with bare hands and well preserved. The sandstones are poorly to moderately sorted, medium-grained, whereas the siltstones are well sorted composed of sub-angular fine-grained sediments ca. 0.5 mm diameter on average. Both lithofacies are horizontally bedded, interbedded with massive siltstone and characterised. The sandstone is slightly porous and absorb water on their surface, but not the siltstones. Fossils such as coral fragments and partly dissolved shells of bivalves, gastropods and plant fragments were discernible from the hand specimen. There was no reaction upon treatment with 3% hydrochloric acid, except where carbonate matter and some fossils occur. According to Tibane et al. [1], primary sedimentary structures were destroyed by bioturbation. Both sandstones and siltstones comprise on average 35% quartz, 20% plagioclase, 5% microcline, 5% mica, <5% siderite, ca. <5% calcite, <5% pyrite, and 5% chlorite and 15% other clay. The siltstone comprises slightly high clay content up to 35% in certain samples.

Planar laminated sandstone B25 (1262.18 – 1262.05)	Sample B25 is light grey to light brown and poorly sorted. It is 2 m thick flat bed with an average 2 mm thick laminations and comprises 1.0 mm diameter grains of 30% quartz, 25% plagioclase, 5% sub-rounded green glauconite grains, 20% clay, <5% narrow flakes of mica, within the matrix consisting of 5% calcite, <5% chlorite, ca. 2% zeolite and ca. 3% pyrite.

Glauconitic sandstone B11 (636.32 – 636.12) B12 (652.27 – 652.17) B39 (1560.88 – 1560.80) Glauconitic siltstone B13 (669.90 – 669.65)	The glauconitic lithofacies of both sandstone and siltstone are mineralogically similar, but only texturally different. Therefore, they were both described together. These samples are greenish to brownish owing to the occurrence of glauconite and chlorite (see section 4.1.1) were observed from uncovered thin sections and dictated by XRD analyses. The lithofacies are well indurated, compacted, massive and hard to break by bare hands. The sandstones are moderately sorted and composed of medium-grained sub-rounded to sub-angular grains. Comparably, the siltstones are moderately to well sorted and comprising fine-grained sediments. Both sandstones and siltstones are characterised by undisrupted bedding planes. They lack bioturbation structures. Plant fragments, leaves and broken shells of animal fossils were observed. Mineralogically, these samples are composed on average 25% angular monocrystalline quartz, 10% sub-angular plagioclase, 10% invertebrate fossils, 10% sub-rounded glauconite, 10% clay matrix, 5% orthoclase, 5% plant fossils, 5% organic matter, and all embedded in 20% calcite cement.
Calcareous sandstone B16 (793.70 – 793.55) B24 (1188.24 – 1188.14) B27 (1336.81 – 1336.65) B28 (1378.00 – 1377.85) B31 (1423.11 – 1422.91) Calcareous siltstone B4 (166.12 – 165.81) B5 (236.22 – 236.12) B6 (287.73 – 287.68) B7 (399.90 – 399.80) B26 (1317.04 – 1316.74)	The calcareous lithofacies of both sandstones and siltstones are combined and described together because they are mineralogically similar, but only textural different. These samples are hard to break by bare hands. They are characterised by indistinct bedding marked by dark seams of organic matter. They are grey owing to the occurrence of carbonate materials. They are porous, poorly sorted and contains lithic clasts of medium-grained size of ca. 0.1 mm diameter on average for the sandstone, whereas the siltstones are moderately sorted and composed of ca. 0.05 mm diameters sediments. These lithofacies are moderately indurated, rich in diverse animal fossils including bivalves, gastropods, cephalopods and small calcareous shell fragments in mm scale. The samples are extensively bioturbated and appear structureless. Mineralogically, these samples are on average 35% sub-angular monocrystalline quartz, 15% twinned angular plagioclase, 15% clay minerals, <5% organic matter (plant fossils) ca. 3% pyrite and 5% 0.3 mm invertebrate fossil cemented in sparry calcite, ca. 2% sub-rounded to rounded glauconite and in 20% poikilotopic fabric of calcite cement. The calcareous sandstone reacts with 3% hydrochloric acid, producing whitish effervescence [1].

Massive carbonaceous sandstone B17 (811.37 – 811.35 m)	The massive carbonaceous sandstone samples are characterised by fine- to medium-grained sediments. They are rich in diverse plant fossils such as rootlets and twigs. This lithofacies is on average consisting of 50% quartz, 20% plagioclase, 10% organic matter, 10% hematite, 5% smectite, and about <5% mica. The massive carbonaceous sandstone represents deposition in shallow marine settings or delta. Preservation of the plant fossils suggests that the deposition was rapid in anoxic environment.

Clast-supported Conglomerate B32 (1426.46 – 1426.36) Matrix-supported Conglomerate B33 (1447.80 – 1447.72) B34 (1453.29 – 1448.16)	Clast supported conglomerates are grey to brownish, composed of sub-rounded to sub-angular clasts of sandstones, metamorphic and volcaniclastic rocks. The samples characterised by red jasper that crystallised following the cracks within the rock. This lithofacies is massive, structureless and consists of poorly sorted and randomly orientated pebble up to 5 cm diameter embedded in sandy matrix. It comprises on average 40% quartz, 25% plagioclase, 5% microcline, 10% siderite, ca. 5% calcite, 10% chlorite and 5% other clay minerals. Matrix-supported conglomerates are comprising pebble embedded in fine to medium-grained sandy matrix. This lithofacies is mineralogically similar to the clast-supported conglomerates

Ash Tuff B38 (1511.50 – 1511.40)	The ash tuff lithofacies such sample B38 occurs in ZB drill core and it is characterised by sub-angular clasts composed of large feldspar in mm-scale and visible to the naked eye. The tuff is greyish dark owing to the plagioclase and quarts. It appears massive and comprising grey to light grey fine-grained ash materials. Petrographic examination of the tuff indicates on average 30% plagioclase and 30% quartz, 20% fine-grained clay, and 20% whitish ash matrix, with some identifiable organic material. The here described tuff lithofacies is moderately to well sorted distal air fall deposit.

Breccia B36 (1483.46 – 1483.34)	Tuff breccia was identified in borehole ZB and it is composed predominantly of dense and dark, altered fragments of igneous rocks compared to surrounding pyroclastic lithofacies. It is characterised primarily by clast sized lithic fragments of ca. 50 mm in diameter and ca.30 mm lapilli-sized pumice, all hosted within the matrix that is composed of fine-grained volcanic ash. Sand sized grains of ca. 0.3 mm sub-rounded orthoclase, 0.2 mm tabular plagioclase and 0.2 mm angular quartz occur scattered within the matrix. Oddly dense and black, altered materials occur and surrounded with plagioclase, quartz and large angular clasts of ca. 50 mm in length occur amongst smaller lapilli sized scoria (ca. 30 mm diameter) and lithic fragments hosted in a matrix composed of fine-grained volcanic ash and black glassy materials of fine zeolite. The breccia has veins that were formed after deposition and are now filled with chalcedony and surrounded by unidentified grey minerals, possibly formed by hydrothermal alteration. Lithic fragments of clast-sized constitutes about 50%, the matrix composed of ash make up to 40%, whereas sand size plagioclase and orthoclase together with clasts of quartz constitute 10%. The tuff breccia was formed from rock fragments from volcanic eruptions and welded together by fine-grained pyroclastic materials.
Porphyritic Rhyolite B37 (1492.30 – 1492.20)	Porphyritic rhyolite with quartz vein cutting through the lava. The lava is hard and difficult to break even using a geological hammer. Sample B37 comprises coarse-crystalline, dark grey to pinkish coloured phenocrysts of feldspar. The sample is massive, contains no organic matter or fossils and no reaction with 3% concentrated hydrochloric acid. The porphyritic rhyolite is consisting of intergrown polycrystalline quartz, feldspar, mica and chlorite. Euhedral coarser white crystal up to 0.5 mm length, particularly quartz crystals occur in contact and touching 0.5 mm grey plagioclase. Brownish, about 0.2 mm long biotite with moderate relief, anhedral and platy with perfect cleavage occur in contact with quartz. This lithofacies is composed of ca. 47% quartz plagioclase and K-feldspar as the common components in ZB averaging 22% and 6% respectively. Chlorite content average is <5%, <5% clay minerals, ca.2% for each of zeolite, pyrite and mica. The content of calcite is 12% on average. The porphyritic rhyolite was formed from volcanic activities during rifting. The crystal size suggest that the porphyritic rhyolite was formed under slow cooling conditions allowing the crystals to growth coarser.

Fossils of gastropod, brachiopod, allochem, and benthic cephalopod were identified from the sediments (Fig. S1) in the raw data, see the link in specifications table. Elongated and calcified echinoderms were embedded in calcite cement containing several clay minerals. Coquina, bivalves and brachiopods are often broken down into numerous fragments to produce sand and gravel-sized particles. Broken oyster and snail shells (snail/mussels) and sand-sized particles were identified. Most of the corals about 2 cm in diameter were often present in an outward radiation pattern. Fig. S1 (A) shows intact gastropod and brachiopod shells, (B) – allochem and longitudinal section of a benthic cephalopod, (C) – calcified elongated echinoderms embedded in calcite cement with some clay minerals, (D) – coquina, bivalves and brachiopods often broken apart into numerous fragments to produce sand and gravel size particles, (E) – intact shells of bivalves, (F) – Oyster, (G) – broken and intact shells of gastropod (snail/turbinate), (H) – broken gastropods shells form sand size particles and a mixture of broken and intact shells of bivalves, and (I) – large component of coral of ca. 2 cm diameter often with outward radiating patterns.

### 1.3 Spectral core imaging

Fig. 1 shows ZB lithological log created from geological core logs and spectral scan data. IntelliCore software was used to provide lithological and mineralogical core data collected from core tray 85-91. The total length of ZB is 1600 m and the top 71.58 m was missing. To examine the variation of minerals with depth, mineral frequencies were plotted against borehole logs and the data show high clay content at 850 m and shallow depths (Fig. S2). Clay minerals were also confirmed by the Red-Green-Blue (RGB) image (Fig. S3) representing chlorite > smectite > kaolinite > illite and later supplemented by XRD data. Illite accounts ca. 75% of all clay minerals. Carbonate content recorded highest at 1400 m and 100 m where limestone occurs, but lowest at 1602-1500 m where volcanic rocks occurs, see the Mineral Index column (Fig. S2; Fig. S4).

Fig. S2 also shows the distribution of sulphates, organics, and clay minerals at various depths through the ZB stratigraphic column. Gypsum occurs at a depth of 1400 m, while Al-smectite and Mg-smectite have been detected in in siliciclastic glauconite bearing siltstone. The proportion of Al-smectite is generally below 5% (Fig. S2), except at depths of 150-100 m, where there is a clear peak that correlates with glauconitic mudstone. Al-smectite is basically absent where limestone facies are present, roughly at 70 m and shallower depth. Mg-smectite occurs at about at 700 m depth, whereas Al-smectite occurs at shallower depths, about 150 m. The higher Mg-smectite content at a depth of about 700 m corresponds to the occurrence of glauconitic siltstone at the same depth. Abundant smectites were also detected by XRD analysis and observed during petrological surveys dispersed within the siltstone facies matrix. A slightly higher chlorite content was seen at a depth of ca. 1500 m and characterised by a clear peak that correlates with weathered volcanic lithoclasts (Fig. S3). Fig. S2 illustrates the different mineral phases and their compositional variations with depth for borehole ZB as measured by spectral method. The graphs show measured clay minerals, carbonate minerals, and organic matter at 500 m, 600 – 500 m, and 71 m. Gypsum occurs at the top of the log around the depth of 400 m – 100 m and it is an alteration product resulting from weathering of the core after 50 years of the core exposure to the atmospheric conditions. Clay mineral content is high towards the top around 700 – 71 m. Carbonate minerals are also concentrated towards the top of the log, from 800 – 71 m. The last column in blue shows the combination of the individual mineral phases.

Fig. S3 provides spectral imaging data of tray 68 at the depth of 652.58 m to 642.82 m in borehole ZB showing the RGB extracted image of the core. The RGB images highlight the distributions of various clay minerals across the drill core. The blue colour demonstrates magnesium smectite, illite is shown in red, and kaolinite is displayed in orange, whereas chlorite is in green.

Fig. S4 shows the occurrence of hydroxides, Fe_carbonate and Fe silicate minerals and their compositional changes at borehole ZB depths from 1600 m to 71.58 m. The Fe_carbonate content increases upwards and is the lowest in 1500 m depth where igneous rocks occur, supported by Fig. 1. High Fe_carbonate content occur in B27 (1336.65-1336.55 m) and B16 (793.70-793.60 m) in calcareous sandstone, in calcareous siltstones such as B30 (1406.65-1406.45 m), B26 (1316.74-1317.04 m), B7 (399.90-399.90 m), B6 ​​(287.73-287.68 m), and also in carbonate rocks such as B20 (988.16-988.03 m), B15 (738.53-738.38 m), B14 (711.96-712, 01 m), B10 (604.72-604.80 m), B8 (491.64-491.49 m), B3 (101.19-101.04 m), B2 (73.76-73.66 m), B1 (71.93-71.58 m).

Fe_silicate content is high in sandstone and siltstone, decreased in calcareous facies and lowest in limestone facies. High Fe_silicate content is measured at a depth of 1500 m where glauconitic sandstone and conglomerate facies are present. This tendency is the opposite of that of Fe_carbonate. Fe_carbonate is a component of the limestone units, which were identified at the depths less than 100 m (e.g., B1, B2, B3) and characterised by the lack of glauconitic sandstone and conglomerate facies. The rapid rise of H_2_O-AlOH occurs at a depth of about 1020 m and a depth of 1400-1500 m, where a sandstone layer has been confirmed. AlOH-MgOH content increased in the borehole, and higher content was measured at depths of 1560 m to 1160 m where glauconitic sandstone and siltstone lithofacies occur. Hydroxides of AlOH-FeOH, H_2_O-FeOH, H_2_O-MgOH, MgOH-FeOH, and AlOH-MgOH show similar trends to Fe_silicate, their content increases with depth with minimum deviations from the surface (Fig. S4). Therefore, the tendency of both hydroxides and Fe_silicate counteracts the tendency of Fe_carbonate of decreasing with depth. AlOH_FeOH, H_2_O_FeOH, H_2_O_MgOH and MgOH_FeOH occur together in sandstone and siltstone, which are dominated in the matrix by glauconite, smectite, mica and chlorite. In contrast, calcareous sandstone, calcareous siltstone, and carbonate rocks are composed of AlOH_MgOH, which is significantly reduced by about 2 times at a depth of 980 m where a calcareous sandstone layer has been confirmed. AlOH_MgOH was occurring together with Fe_carbonate.

Fig. S4 illustrates hydroxides, Fe_carbonate and Fe_silicate minerals and their compositional variations from the depth of 1600 – 100 m for borehole ZB. Fe_carbonate content decreases with increasing depth. The content of Fe_silicate, H2O_AlOH and AlOH_MgOH decrease greatly upwards, whereas the content of AlOH_FeOH, H_2_O_FeOH, H_2_O_MgOH, MgOH_FeOH and MgOH1_MgOH_2_ is decreasing relatively constant. A sharp decrease of AlOH_MgOH content occurs at ca. 980 m depth at a calcareous sandstone bed. These distributions are important for the evaluation of the suitable lithofacies for the purpose of CO_2_ sequestration.

### 1.4 Scanning electron microscopy

Fig. S5 shows the SEM data before and after processing the sample with CO_2_. The feldspar before CO_2_ treatment is shown in Fig. S5A together with the detrital plagioclase (2b), and the feldspar after the reaction (Fig. S5B) is shown as 2a (albite), which is experimentally formed. The detrital quartz (1b) reacted with scCO_2_, as shown by the eroded edges, and produced surface roughness (1a). Dissolution pits and degraded jagged grain edges can be seen on the quartz (Fig. S5B). The plagioclase in Fig. S5A is in contact with quartz before reaction, but after the reaction, the particles are separated (Fig. S5A). Mica (3a) showed reaction after treatment with scCO_2_.

Fig. S5 (A) shows SEM photomicrographs of an arkosic sandstone sample from drill core ZB before CO_2_ treatment, comprising grains of quartz (1b), plagioclase (2b), and mica (3b). The mineral grains are connected to each other (1b in contact with 2b). (B) – illustrates B18 after CO_2_ treatment, plagioclase grains (2a) show corrosion, dissolution features with pitted surface topography, and separated from quartz (1a), creating intergrain porosity. 3a represents mica after CO_2_ treatment.

EDS data for plagioclase (2b) before and feldspar (2a) (albitisation) after CO_2_ treatment of sample B18 of the arkosic sandstone reported in weight percentages (wt.%), indicates introduction of K, an increase of Fe and Ca, accompanied by a decrease of Na, Al, Mg, Si, and O (Table S1). Table S1 provides EDS data for plagioclase (2b) before CO_2_ treatment and feldspar (2a) (albitisation) after CO_2_ treatment of the arkosic sandstone (sample B18) measured in weight percentages (wt.%). The composition of biotite before and after CO_2_ treatment introduction of K, an increase of Fe, Ca, accompanied by a decrease of Na, Al, Mg, Si, and O.

### 1.5 X-ray diffraction (XRD) data

Mean mineralogy data by wt% of arkosic sandstone reported using the XRD method (Section 2.5) is: quartz (35.75) > clays (23.75) > plagioclase (22.5) > calcite (6.25) > zeolite (4.5) > chlorite (2.86) > pyrite (2.63) > mica (2.63) > microcline (2) > siderite (0.00) (Table 3), reflecting higher silicates and clay content. The average mineralogy of argillaceous siltstone, expressed in wt%, is comparable to that of arkosic sandstone. In contrast, the average mineralogy by wt% of calcareous sandstone is mainly quartz (33.83) > siderite (22.00) > calcite (20.00), plagioclase (15.83) > clays (11.33) > chlorite (6.40) > zeolite (4.00) > mica (2.80) > microcline (2.00) > pyrite (1.60) (Table 3). Calcareous siltstone is mineralogically similar to calcareous sandstone. Compared to arkosic sandstone and argillaceous siltstone, calcareous siltstone and calcareous sandstone are characterised by high carbonate, chlorite and mica content on average. In general, the content of quartz, plagioclase, clay, zeolite and pyrite in siliceous samples is slightly higher than in calcareous samples.

**Table 3:** Mineralogical analyses of forty samples collected from borehole ZB and measured in weight percentage (wt.%), by using X-ray diffraction techniques.

		Depth (m)												
Lithofacies	Sample ID	From	To	Calcite	Siderite	Pyrite	Microcline	Plagioclase	Quartz	Mica	Chlorite	Zeolite	Clays	Total
Bioclastic limestone	B1	71.93	71.58	97	-	-	-	-	3	-	-	-	-	100
	B2	73.76	73.66	99	-	tc	-	-	tc	-	-	-	-	99
	B3	101.19	101.04	97	-	tc	-	-	tc	-	tc	-	-	97
	B8	491.64	491.49	73	-	tc	tc	11	8	tc	tc	2	2	96
	B10	604.80	604.70	57	-	2	tc	10	16	tc	-	3	9	97
	B14	712.01	711.96	55	-	1	tc	13	12	tc	tc	3	13	97
	B15	738.53	738.38	50	-	2	tc	13	19	tc	tc	tc	12	96
	B20	988.16	988.03	66	-	tc	tc	8	16	2	tc	2	2	96
	B30	1406.65	1406.45	40	11	tc	tc	5	30	2	5	tc	4	97

Calcareous sandstone	B16	793.70	793.55	32	-	1	tc	10	45	-	-	6	4	98
	B24	1188.24	1188.14	13	-	2	tc	24	29	3	4	2	23	100
	B25	1262.18	1262.05	14	-	3	tc	23	36	4	5	tc	14	99
	B27	1336.81	1336.65	45	-	tc	2	13	29	2	4	tc	3	98
	B28	1378.00	1377.85	10	24	0	tc	12	26	2	8	tc	17	99
	B31	1423.11	1422.91	6	20	2	tc	13	38	3	11	-	7	100

Calcareous siltstone	B4	166.12	165.81	24	-	3	tc	14	39	4	3	-	13	100
	B5	236.22	236.12	26	-	2	5	8	40	3	2	3	11	100
	B6	287.73	287.68	43	-	2	tc	15	27	2	tc	4	6	99
	B7	399.90	399.80	44	-	tc	tc	16	24	tc	-	2	11	97
	B26	1317.04	1316.74	19	-	2	tc	21	41	3	5	tc	8	99
Carbonaceous sandstone	B17	811.40	811.35	18	-	4	tc	20	34	2	tc	2	18	98
Clast-supported conglomerate	B32	1426.46	1426.36	-	6	tc	tc	12	61	-	14	-	5	98

Glauconitic sandstone	B11	636.32	636.12	4	-	2	tc	22	42	4	2	-	22	98
	B12	652.27	652.17	5	-	4	3	10	38	21	3	12	4	100
	B39	1560.88	1560.80	2	2	3	2	16	28	6	12	-	30	101
Matrix-supported conglomerate	B33	1447.80	1447.72	2	14	tc	8	31	29	-	8	-	6	98
	B34	1453.29	1448.16	tc	12	tc	9	40	23	tc	5	-	8	97
Porphyritic rhyolite	B37	1492.30	1492.20	tc	17	tc	8	30	38	-	3	-	3	99

Argillaceous Siltstone	B9	551.38	551.28	13	-	3	tc	19	43	tc	tc	tc	17	95
	B19	924.86	924.74	5	-	3	3	21	35	5	3	3	23	101

Arkosic Sandstone	B13	669.90	669.65	8	-	3	tc	15	24	4	3	9	33	99
	B18	869.39	869.29	6	-	3	tc	18	33	2	tc	-	36	98
	B21	1046.99	1046.81	9	-	2	2	17	41	3	3	3	21	101
	B22	1093.32	1093.27	8	-	3	2	22	34	3	3	-	25	100
	B23	1126.39	1126.29	7	-	3	2	23	37	2	2	tc	23	99
	B29	1214.63	1214.48	7	-	3	tc	29	29	3	3	2	23	99
	B35	1470.36	1462.74	2	-	2	tc	30	45	2	2	4	12	99
	B40	1570.12	1570.02	3	tc	2	2	26	43	2	4	-	17	99
Tuff breccia	B36	1483.46	1483.34	5	9	tc	3	4	74	tc	2	tc	-	97
Ash tuff	B38	1511.50	1511.40	5	11	2	7	33	29	2	7	2	3	101

### 1.6 X-ray fluorescence (XRF) data

The XRF data of the major elements (wt%) for arkosic sandstone of borehole ZB are mainly SiO_2_ (58.16) > Al_2_O_3_ (13.21) > Fe_2_O_3_ (9.35) > CaO (4.84) > H_2_O-(4.46) > MgO (3.87) > Na_2_O (2.71) > K_2_O (1.78) > TiO_2_ (1.10) > P_2_O_5_ (0.12) > MnO (0.08) > Cr_2_O_3_ (0.03) and 4.57 wt% of loss on ignition (LOI) (Table 4). The average geochemistry of the argillaceous siltstone is comparable to that of the arkosic sandstone. Contrarily, the average geochemistry (wt%) of the calcareous sandstone is predominantly SiO_2_ (52.67) > Al_2_O_3_ (11.28) > CaO (9.98) > Fe_2_O_3_(t) (8.19) > H_2_O- (3.06) > MgO (2.61) > Na_2_O (1.75) > K_2_O (1.42) > TiO_2_ (0.87) > MnO (0.14) > P_2_O_5_ (0.14) > Cr_2_O_3_ (0.02) with LOI of 11.08 wt% (Table 4).

**Table 4:** Geochemical analyses of forty samples collected from borehole ZB and measured for major elements in weight percentage (wt.%), analysed by using X-ray fluorescence. Carb. = carbonaceous, Cs_cong. = clast-supported conglomerate, and Por. = porphyritic.

Lithofacies	Sample	From (m)	To (m)	SiO_2_	TiO_2_	Al_2_O_3_	Fe_2_O_3_(t)	MnO	MgO	CaO	Na_2_O	K_2_O	P_2_O_5_	Cr_2_O_3_	LOI	H_2_O^-^	Total
Bioclastic limestone	B1	71.93	71.58	9.88	0.05	0.68	0.62	0.014	1.01	59.70	0.04	0.13	0.079	0.003	26.34	1.09	99.64
	B2	73.76	73.66	7.56	0.02	0.49	0.82	0.014	1.06	62.82	0.02	0.06	0.048	0.003	25.68	2.22	100.81
	B3	101.19	101.04	6.74	0.07	1.34	2.29	0.030	4.77	53.76	0.07	0.39	0.241	0.006	28.97	1.74	100.42
	B8	491.64	491.49	18.79	0.37	4.75	3.69	0.473	1.25	40.45	0.90	0.71	0.698	0.010	26.91	1.30	100.30
	B10	604.80	604.70	36.13	0.67	8.56	5.20	0.162	2.11	25.10	1.53	1.23	0.085	0.017	18.97	3.47	103.24
	B14	712.01	711.96	37.34	0.90	9.28	6.81	0.227	2.61	22.82	1.86	0.98	0.095	0.020	16.88	3.63	103.46
	B15	738.53	738.38	37.86	0.75	8.70	6.17	0.178	2.56	22.79	2.06	1.15	0.107	0.016	16.30	2.61	101.24
	B20	988.16	988.03	35.10	0.62	8.00	5.27	0.244	1.70	25.95	1.29	1.47	0.120	0.018	20.15	2.16	102.10
	B30	1406.65	1406.45	44.74	0.79	9.80	6.00	0.069	1.73	16.72	1.09	1.26	0.107	0.022	17.65	3.00	102.97

Calcareous sandstone	B16	793.70	793.55	47.49	0.49	6.12	5.33	0.232	1.67	20.69	1.24	0.69	0.267	0.014	15.86	1.04	101.12
	B24	1188.24	1188.14	57.92	0.99	13.80	8.48	0.074	3.27	5.91	2.57	1.86	0.136	0.025	5.25	3.79	104.09
	B25	1262.18	1262.05	54.06	1.13	14.09	9.40	0.080	4.09	6.07	2.29	1.75	0.125	0.024	6.94	3.75	103.80
	B27	1336.81	1336.65	39.05	0.72	9.49	5.26	0.261	1.92	21.89	1.26	1.35	0.082	0.016	19.03	2.07	102.40
	B28	1378.00	1377.85	58.48	1.01	11.43	10.11	0.077	2.76	2.65	1.90	1.28	0.116	0.024	9.96	4.40	104.19
	B31	1423.11	1422.91	59.04	0.89	12.73	10.54	0.139	1.96	2.63	1.21	1.61	0.112	0.023	9.43	3.46	103.77

Calcareous siltstone	B4	166.12	165.81	53.57	0.81	11.09	6.50	0.048	3.80	9.61	2.20	2.27	0.073	0.020	8.87	4.82	103.69
	B5	236.22	236.12	57.66	0.74	10.50	5.65	0.038	3.32	8.02	1.36	3.86	0.081	0.021	8.11	4.04	103.40
	B6	287.73	287.68	35.83	0.51	7.00	3.64	0.124	1.89	27.55	1.34	0.93	0.067	0.013	21.02	2.64	102.55
	B7	399.90	399.80	51.13	0.72	8.82	5.49	0.071	2.54	15.90	1.97	1.28	0.098	0.023	11.92	3.03	103.01
	B26	1317.04	1316.74	55.08	1.02	13.41	8.12	0.056	3.55	6.61	1.94	2.08	0.121	0.020	8.00	3.44	103.44

Glauconitic sandstone	B11	636.32	636.12	60.68	1.09	13.28	8.67	0.052	3.29	3.54	2.47	2.31	0.104	0.028	4.15	4.39	104.06
	B12	652.27	652.17	55.75	0.69	10.32	16.84	0.036	3.39	1.62	1.15	5.14	0.141	0.031	4.86	5.36	105.35
	B39	1560.88	1560.80	50.43	1.22	10.50	19.28	0.125	5.21	2.74	1.69	2.73	0.204	0.049	4.37	4.33	102.88
Carb. sandstone	B17	811.40	811.35	58.73	1.08	10.66	9.20	0.094	3.10	8.59	2.07	1.19	0.105	0.028	5.16	3.56	103.57
Cs_cong	B32	1426.46	1426.36	68.42	0.47	15.71	2.40	0.062	0.41	1.03	3.63	5.44	0.242	<0.001	2.09	1.35	101.26
Matrix-supported conglomerate	B33	1447.80	1447.72	62.62	0.52	17.28	5.83	0.309	0.22	0.61	4.30	5.98	0.115	<0.001	2.84	0.95	101.58
	B34	1453.29	1448.16	63.20	0.56	17.63	4.29	0.103	0.22	0.65	4.37	6.45	0.124	<0.001	2.57	1.23	101.40
Por. rhyolite	B37	1492.30	1492.20	63.65	0.45	15.06	6.23	0.264	0.22	0.51	4.20	6.32	0.082	<0.001	2.78	0.83	100.60

Arkosic sandstone	B13	669.90	669.65	56.37	1.06	13.87	9.21	0.091	3.95	5.59	3.01	1.77	0.119	0.025	4.64	4.68	104.40
	B18	869.39	869.29	58.04	1.28	12.44	9.52	0.103	4.33	5.22	2.60	1.35	0.105	0.035	4.92	5.38	105.33
	B21	1046.99	1046.81	58.97	1.06	12.38	9.12	0.074	3.86	5.31	2.45	1.89	0.133	0.029	4.70	4.44	104.41
	B22	1093.32	1093.27	58.09	1.07	13.49	9.46	0.077	3.67	4.90	2.56	2.03	0.113	0.028	4.73	3.93	104.14
	B23	1126.39	1126.29	58.23	1.02	13.75	8.94	0.083	3.63	5.19	2.82	1.77	0.112	0.026	4.73	3.88	104.19
	B29	1214.63	1214.48	56.69	1.06	13.58	9.81	0.071	3.71	4.85	2.45	1.75	0.125	0.028	5.34	3.92	103.39
	B35	1470.36	1462.74	58.08	1.16	14.35	9.94	0.095	4.09	4.01	2.95	1.60	0.116	0.029	3.86	6.61	106.88
	B40	1570.12	1570.02	60.83	1.05	11.83	8.80	0.078	3.69	3.66	2.82	2.05	0.127	0.026	3.63	2.82	101.39

Argillaceous siltstone	B9	551.38	551.28	55.24	1.01	13.00	8.37	0.063	3.59	6.95	2.30	1.90	0.111	0.025	7.20	4.35	104.12
	B19	924.86	924.74	58.14	1.04	14.19	9.29	0.055	3.38	4.08	2.46	2.14	0.120	0.025	4.96	3.74	103.63
Tuff breccia	B36	1483.46	1483.34	78.10	0.15	5.10	6.44	0.144	0.21	2.23	1.19	1.89	0.033	<0.001	4.17	0.47	100.12
Ash tuff	B38	1511.50	1511.40	63.45	0.48	16.05	5.57	0.208	0.18	1.29	4.83	5.91	0.075	<0.001	1.44	0.37	99.86

**Fig. 2:**
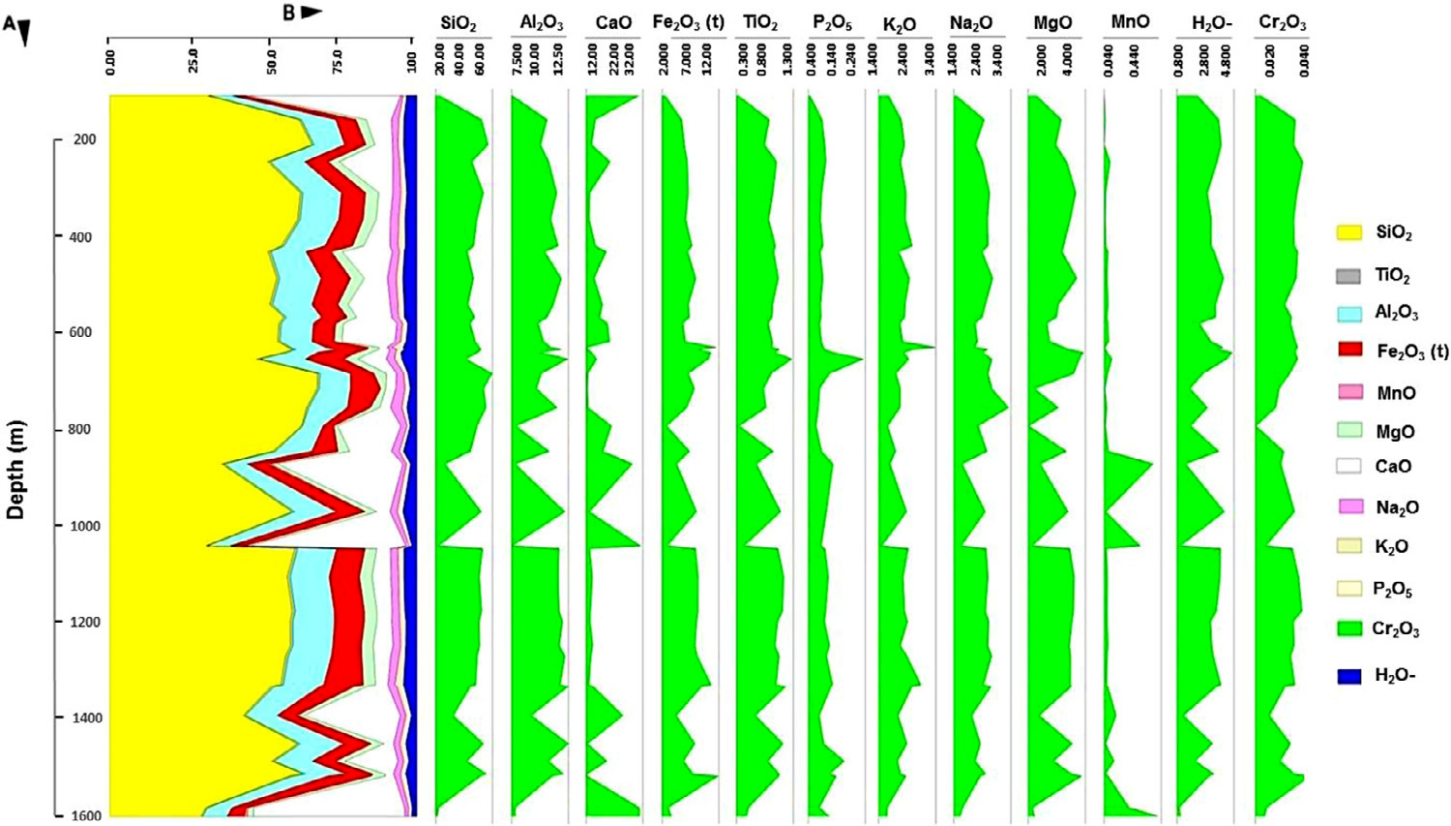
Vertical distribution of major elements recorded using XRF method and reported in weight percentage (wt.%) for lithological samples of borehole ZB. High CaO content occurs at 1600 m, 1050 m and at 150-70 m depth, whereas high MnO content was recorded at 1600 m and between 1050 and 900 m depth. Contrarily to CaO, SiO_2_ and all other oxides demonstrate reversed trends especially at the depth of 1050 m.

Calcareous siltstones are geochemically similar to the lithology of calcareous sandstones. The geochemistry of the arkosic sandstones and argillaceous siltstone contain on average high SiO_2_, Al_2_O_3_, Fe_2_O_3_, MgO, Na_2_O, K_2_O, TiO_2_, and Cr_2_O_3_, while the calcareous siltstone is defined by high average content of CaO, P_2_O_5_, and MnO. This trend corresponds to XRD data (Section 1.5). However, it is recognised that the XRF data contains restrictions on the distinction between clay minerals and mica, as well as the distinction between clay minerals. Nevertheless, the XRF data was supplemented by EDS spectral data (Section 1.4) and spectral scan data (Section 1.3). Fig. 2 displays the vertical distribution of the major elements recorded by the XRF method in wt.% for borehole ZB lithological samples. Elevated CaO content occurs at the depth of 1600 m, 1050 m and 150-70 m, whereas high MnO content was recorded at the depth of 1600 m and 1050 – 900 m. In contrast to CaO and MnO, SiO_2_ and all other oxides show a reversed trend at the depth of 1050 m. The opposite trend between CaO and SiO_2_ corresponds to the observations made on the spectral scan data (Section 1.3).

Arkosic sandstone (A20, A24, A29, B18, B21, B40, C8, C13, C25, C38 and N32), calcareous sandstone (A21, B16, B28, C3 and N25), argillaceous siltstone (A27, A31 and A38), calcareous siltstone (A12) porosity data was obtained by examining the intraporosity of quartz, plagioclase, k-feldspar, hematite, calcite, siderite, and clay minerals, before and after treatment with scCO_2_. The data show that the unreacted arkosic sandstone and argillaceous siltstones are more porous, whereas the reacted calcareous sandstone and calcareous siltstones are less porous (Table 5).

**Table 5:** The intraporosity was recorded in percentage (%) determined by using semi-quantitative 2D image analysis using uncovered thin sections impregnated with blue epoxy using samples of arkosic sandstone, argillaceous siltstones, calcareous sandstone and calcareous siltstones before and after treatment with scCO_2_.

	Intraporosity of the minerals in percentage (%), before and after treatment with scCO_2_							
	Arkosic		Calcareous		Argillaceous		Calcareous	
	sandstone		sandstone		siltstone		siltstone	
	A20, A24, A29, B18							
	B21, B40, C8, C13		A21, B16, B28,		A27, A31			
	C25, C38 and N32		C3 and N25		and A38		A12	
Mineral Phases	Before	After	Before	After	Before	After	Before	After
Quartz	-	-	-	-	-	-	-	-
Plagioclase	1	4	1	4	1	4	2	4
K-feldspar	2	3	1	2	2	3	1	2
Hematite	1	1	1	2	1	1	2	2
Calcite	2	2	1	2	1	1	1	3
Siderite	1	1	1	2	2	1	1	1
Clay	1	2	1	3	2	2	1	2

Stress levels varied significantly in both directions for all four types of rock examined. The data show that the uniaxial compressive strength of arkosic sandstone ranges from 6 MPa in the vertical direction to 15 MPa parallel to the bedding plane. For comparison: calcareous sandstones are 3.40 MPa to 4.80 MPa perpendicular and parallel to the bedding respectively. The lithological strength depends on the direction of the load, but the data show that the arkosic lithologies are significantly stronger than their counterparts calcareous sandstones. Nonetheless, both silicate and calcareous lithologies withstood the forces acting vertically on the bedding and broke easily when the forces were applied parallel to the stratification (Table 6). In addition, the data showed that the strength of the material tested was controlled by the chemical composition. There is a correlation between the amount of carbonate and silicate content to the strength of the rocks. Arkosic sandstone is composed of calcite (4.44), quartz (34.45), clay (25.82) and feldspar (23.82) on wt% averages (Table 3), recording a maximum of 15 MPa (Table 6), but calcareous sandstone is composed on average wt% calcite (28.60), quartz (12.60), clay (13.00), feldspar (36.00), recording up to 4.8 MPa. In general, the data show that arkosic sandstone is twice as strong as calcareous sandstone.

**Table 6:** Average measurements of the compressive stress in two dimensions where σ_1_ is the principal stress perpendicular to the bedding planes, whereas σ_2_ is the principal stress parallel to the bedding of the original sandstone and siltstone facies.

Strength Tests							
		Stress	Distance	Force	Is	Pressure	UCS = 24Is
Lithology Type	Sample	Direction	(mm)	(N)	(50)	(MPa)	(50)
Arkosic sandstone	C8, A20, A24, A29, B18, B21, B40, C13, C25, C38	σ_1_	25.2	5400	9	6.00	144
		σ_2_	17.1	7400	26	15.00	360
Calcareous sandstone	N25, N32, A21, B16, B28, C3, B16, B24, B25, B27, B28, B31	σ_1_	20.5	2600	6	4.00	96
		σ_2_	37.0	5400	4	3.40	82
		σ_1_	22.0	2200	5	3.50	84
		σ_2_	21.0	3200	7	4.80	115
Argillaceous siltstone	A31, A27, A38, B9, and B19	σ_1_	16.1	2800	11	7.00	168
		σ_2_	23.5	3400	6	4.00	96
Calcareous siltstone	A12, B4, B5, B6, B7, B26	σ_1_	15.5	2600	11	6.80	163
		σ_2_	21.5	3200	7	4.80	115

On average wt%, the argillaceous siltstones are consisting of calcite (8.00), quartz (24.00), plagioclase (15) and clay (33.00). The UCS ranges from 4 MPa parallel stratification to 7 MPa perpendicular to stratification. In contrast, the stress value perpendicular to the stratification is 4.80 and 6.80 MPa along the stratification plane for calcareous siltstonewith averages wt% of calcite (27.50), quartz (36.67), plagioclase (13.67) and clay (9.83). Since siltstone is composed of fine-grained sediments and is less porous than sandstone, no significant difference in stress levels was observed between argillaceous siltstone and calcareous siltstone, but the data show that the siltstones had different stress levels based on the direction in which the stress was applied, and also based on chemical composition differences.

### 1.7 Fluid-rock interactions

**Fig. 3:**
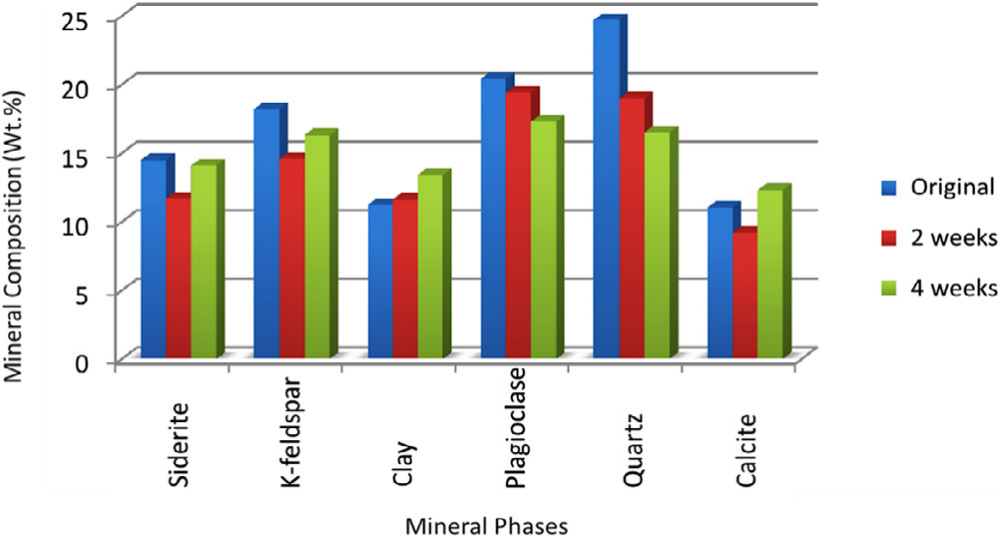
Comparisons of mineral content between arkosic sandstone samples that were not reacted with CO_2_ and samples that were treated for two weeks and those samples that were treated for four weeks. The samples were analysed using XRD technique and the data are reported in weight percentage (wt.%).

Both sandstone and siltstone samples respond to scCO_2_ [1]. Feldspar, quartz and carbonate cement dissolved during the interaction of H_2_O–CO_2_–rocks (Fig. 5B and D). Fig. 3 shows the mineral composition before and after treating the sample with scCO_2_. The porosity and permeability of the reacted sandstone and siltstone changed due to the geochemical interaction with scCO_2_. The original arkosic sandstone porosity was below 25% before CO_2_ treatment and 40% after scCO_2_ treatment, whereas the permeability was between 0.79 and 1.0 mD before and after scCO_2_ treatment respectively [1]. Siltstone porosity was below 5% before treatment with CO_2_ and slightly above 5% after treatment with CO_2_, whereas the permeability remained below 0.79 mD [1].

### 1.8 Properties of the sandstone and siltstone samples

The sandstone and siltstone lithologies investigated are layered, fine to medium-grained, with low porosity of less than 10% and permeability of less than 1.0 mD [2]. All lithologies were relatively soft, exhibited uniaxial compressive stresses of 6 – 15 MPa and reacted with scCO_2_ after four weeks (Table 7).

**Table 7:** Lithological information, mineralogy, porosity and permeability, geomechanical testing, and CO_2_-H_2_O-rock interaction.

Lithology	Porosity	Permeability	Mineralogy	UCS	Structures	Observations
Arkosic sandstone	Low, 5-10%	Low permeability, <10 md [2]	>55% quartz, 20% plagioclase, 15% clay minerals, <5% carbonates	6-15 MPa	Bedded	Reacted with scCO_2_
Calcareous sandstone	Low, < 10%	Low permeability, <10 md [2]	<40% quartz, ca. 25% feldspar, 15% clay minerals, ca. 20% carbonates	4.0 MPa average	Bioturbated	Reacted with scCO_2_
Siliceous siltstone	Low, < 10%	Low permeability, <10 md [2]	Ca. 40% quartz, ca. 25% feldspar, 15% clay minerals, and 20% carbonates	5.5 MPa average	Laminated	Reacted with scCO_2_
Calcareous siltstone	Low, < 5%	Low permeability, <10 md [2]	<40% quartz, >30 carbonates, >15% feldspar, >15% clay minerals	5.8 MPa average	Bioturbated	Reacted with scCO_2_

## 2 Experimental Design, Materials and Methods

### 2.1 Spectral imaging

**Fig. 4:**
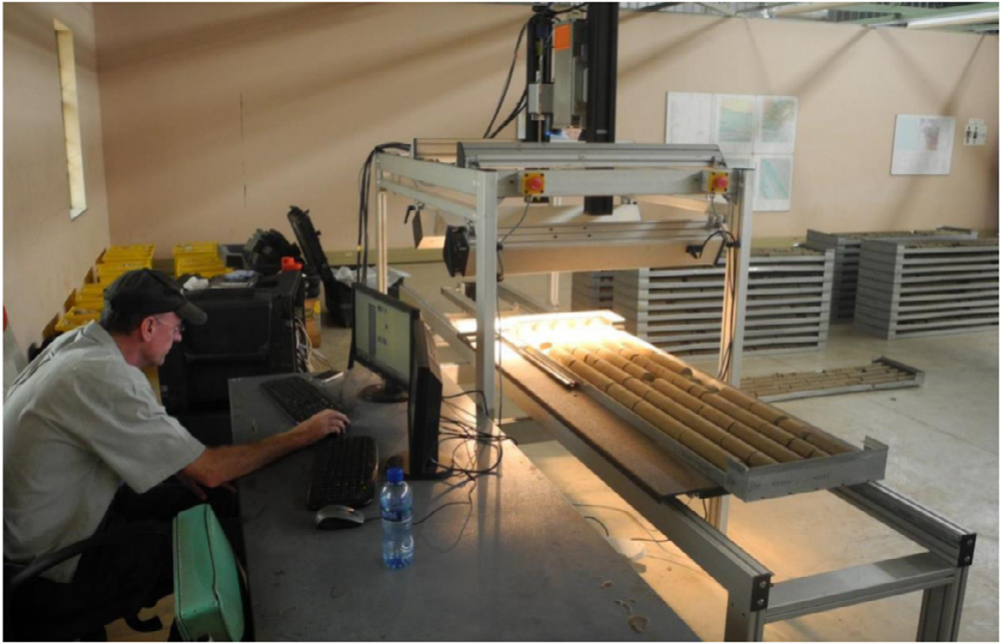
sisuMOBI spectral imaging machine in operation at the National Core Library at Donkerhoek in Pretoria.

The sisuRock mobile spectral imaging device (sisuMOBI) was used to scan the Zululand basin NZA, ZA, and ZB drill cores quickly and accurately (Fig. 4). Spectral imaging technology is a passive, non-destructive technology with the ability to objectively and consistently measure and record core data. The three drill cores were completely scanned within four days at Donkerhoek National Core Library. This method was chosen because it can perform ex situ measurements and therefore requires minimal sample preparation. The spectral scanning data are reliable and comparable to XRD data.

To avoid varying degrees of reflection that could distort measurements from the surface of the minerals, the cores were cleaned to remove the dust particles that covered the original mineral. The cores were then dried overnight. Using a series of image sensors operating in different regions of the visible and infrared electromagnetic spectrum [3], the cores were spectrally scanned to enable mineral detection in red (620-750 nm), green (495-570 nm) and blue (450-495 nm) wavelength. Short-Wave Infrared (SWIR) in the 400-2500 nm range, Visible-Near Infrared (VNIR), Mid-Wave Infrared (MWIR), and Long-Wave Infrared (LWIR). Measurements were recorded from the surface of the sample with a penetration depth of 0.4-14 µm (Table 8). The instrument took infrared images of the cores with a spatial resolution between 0.1 mm and 0.5 mm in the SWIR range and operated at detection speeds measured in seconds.

**Table 8:** Approximate ranges of the different infrared wavelengths in microns.

Region	Infrared range	Code	Approximate range (µm)
Visible	Visible Near Infrared	VNIR	0.4 – 1.0
Near Infrared	Short-Wave Infrared	SWIR	1.0 – 2.5
Mid-Infrared	Mid-Wave Infrared	MWIR	3.0 -6.0
	Long-Wave Infrared	LWIR	6.0 -14.0

Manipulating the spectrum scan method in a wavelength field of 0.4-2.5 μm was able to analyse and record iron minerals (goethite), clay minerals, hydroxyl groups and carbonates (Figs. S2 and S4) [3]. Images include colour images, feature extraction images, mineral presence maps (Fig. 1). Infrared images and digital geochemical data provide textural and mineralogical information about the drill cores. Visual images are important for displaying mineralogical changes, distribution, abundance, and spectrum of minerals that reflect lithological changes. In contrast, digital geochemical data includes mineral indices, mineral counts, statistical data, and graphs used to interpret and compare geology within the ZB core, NZA, ZA, and ZC.

VNIR's mineral detection capabilities are limited. Nevertheless, it was used to identify hydroxyl and carbonate measured in the SWIR, MWIR, and LWIR ranges. Clay minerals, mica, chlorite, and carbonates (e.g., aragonite and calcite) have been identified throughout the SWIR region. The most important basic absorption properties associated with OH bonds in minerals are in the MWIR range. A longer wavelength range was used to detect the basic vibrations of silica and aluminium. Pyroxene, quartz, feldspar, sulphate, and other silicate minerals were detected and identified by thermal infrared (LWIR in the 713 µm range. These could not be previously measured with the SWIR spectrum alone when collecting data.

### 2.2 Geological core logging

Ten exploration drill cores, stored at the National Core Library in Pretoria, were physically inspected and cleaned to remove dust from their surface. Uncleaned cutting material exists at intervals of 1.5 m from the four drill holes including ZD 1/71 (324.61 m), ZE 1/17 (579.12 m); ZF 1/72 (527.30 m); and ZG 1/72 (461.77 m). The core ZD1/71, about 29.87 m long, consisted of five boxes with depths of 174.04 m to 558.70 m, and about half of the core consisted of intrusive rocks. The drill cores ZE1/17, ZD 1/71, and ZG 1/72 were heavily fragmented and did not have the intact hard material needed for sampling to prepare the thin sections. These drill cores contained intrusive rocks, which made it difficult to cut the rocks in preparation for thin sections and were considered unsuitable for further investigation. Relatively well-preserved core materials were present in the ZA, ZB, ZC, and NZA drill cores (Table 1). The ZA was about 1600 m long and was stored in 200 core boxes. Box 4 and box 5 were missing at the depth of 98.87-100.58 m. ZC was about 2000 m stored in 220 core boxes. NZA was recorded in roughly depth 579.12 m, stored in 31 core boxes. Boreholes ZA, ZB, ZC, and NZA cores were cleaned, recorded, and photographed. The drill core logs were recorded while measurements were taken at intervals where significant changes in lithology were observed in vertical sections.

### 2.3 Sample collection

Sampling was carried out between January and April 2014. Samples were taken at random intervals or when there were major changes in vertical lithology. Emphases were placed on sedimentary lithology that could function as reservoirs or caprocks. Using a cutting saw, the cores were split into samples about 15 cm long. The drill core samples were inspected in the laboratory using a combination of different analytical techniques to validate the field description. Samples were described by rock type, colour using Munsell(@ rock colour chart, sediment structure, grain size, grading, roundness, mineral type, fossil generation and type. A total of 40 rock samples were collected from ZB, 40 samples from ZA, 40 samples from ZC and 40 samples from NZA. Samples were marked A for drill hole ZA, B for drill hole ZB, C for drill core ZC, N for NZA, and numbered from 1 to 40 (Table 2).

### 2.4 Petrography

The texture and compositional properties of the lithology investigated were based on both macroscopic (Fig. S1) and uncovered thin sections viewed under scanning electron microscopy (Fig. S5). Due to the presence of siltstone covering sandstone, special attention was focused to samples taken below 800 m, representing the minimum preferred depth required for CO_2_ storage. A total of 160 uncovered thin sections were prepared at the Council for Geoscience and investigated by using a Nikon Eclipse transmitted light microscope available from the University of Pretoria. The petrographic data of the thin sections provided a baseline that was accepted to determine the various lithologies. Lithology and associated mineralogy, particle size, grade, shape, and colour helped determine the depositional environments of sediments and carbonate deposits. Sedimentary structures, digenetic features, fossils, and trace fossils have also been investigated to help deduce sedimentary environments. Microfacies examinations including matrix, particle size, fossils and their content, and facies zones were correlated between boreholes. A total of 18 thin sections were stained with blue epoxy and used to determine the type of porosity and permeability. Assessments of the porosity and permeability of sandstone and siltstone selected for CO_2_ storage were carried out at the Department of Geosciences and Geography of the Martin-Luther University, Halle-Wittenberg, Germany.

### 2.5 X-ray diffraction (XRD)

For bulk composition analysis, 160 representative whole rock samples from ZA, ZB, ZC and NZA were crushed, milled and homogenized to powder material at UP. The milled powder samples were sent to the CGS where a McCrone micronizing mill was used to reduce the particle size to approximately 5 to 10 µm. A sub-sample was taken and pressed into a shallow plastic sample holder against a rough filter paper ensuring random orientation. Mineral identification was performed on a Bruker D8 Advance X-ray Diffraction (XRD) instrument with 2.2 kW Cu long fine focus tube (Cu Kα, λ=1.54060) and 90 position sample changers. The system is equipped with Lynx Eye detector with 3.7° active area. Samples were scanned from 2 to 70° 2θ at a speed of 0.02° 2θ steps size per 3 seconds, and generator settings of 40 kV and 40 mA. Phase identification was based on BRUKER DIFFRAC^Plus^ - EVA evaluation program. The ICDD (JCPDS) Inorganic/Organic Data base was used for phase search, of which the PDF-2 Release 2006 was available. Quantitative XRD analyses by Rietveld method were performed using DIFFRAC^Plus^ – TOPAS software with accuracy in the region of ±1%. The structures of the components are generated from structure databases such as the Inorganic Crystal Structure Database (ICSD), the Cambridge Structure Database (CSD) or TOPAS Structure Database provided by the instrument/software supplier. The quantification of the amorphous phase was done by adding ZnO as an internal standard. This method was adapted from British Standard: BS EN 13925-2003 and implemented to suit the specifics of CGS XRD Laboratory. This method was chosen for mineral detection because its high resolution ensured that peak positions were more accurately determined, and mineral phases were better resolved and quantified. The Rietveld method is an ultimate tool for mineral identification and volume estimation from powder diffraction data. The observed diffraction pattern is assumed to be a sum of background function and contributions from all individual minerals. Once individual observed peak intensities are extracted, they are compared to the calculated peak intensities obtained from the crystal structure for the individual mineral.

### 2.6 X-ray fluorescence (XRF)

For major element analysis, 160 samples from ZA, ZB, ZC and NZA were milled at UP down to <75 µm fraction and sent to CGS where they were roasted at 1000 °C for at least 3 hours to oxidise Fe^2+^ and S and to determine the loss of ignition (L.O.I). Glass disks were prepared by fusing 1g roasted sample and 9.5 g flux consisting of 70.689% Li_2_B_4_O_7_, 19.786% LiBO_2_ and 0.50% LiI at 950 °C. For trace element analysis 12 g milled sample and 3 g Hoechst wax were mixed and pressed into a powder briquette by a hydraulic press with the applied pressure at 25 ton. The glass disks and wax pellets were analysed by a PANalytical Axios X-ray fluorescence spectrometer equipped with a 4 kW Rh tube. XRF technique is most suitable to identify and quantify the major oxides of the examined samples milled down to less than 5 µ fraction.

### 2.7 Geochemical modelling

#### 2.7.1 Water-CO_2_-rock interaction

**Fig. 5:**
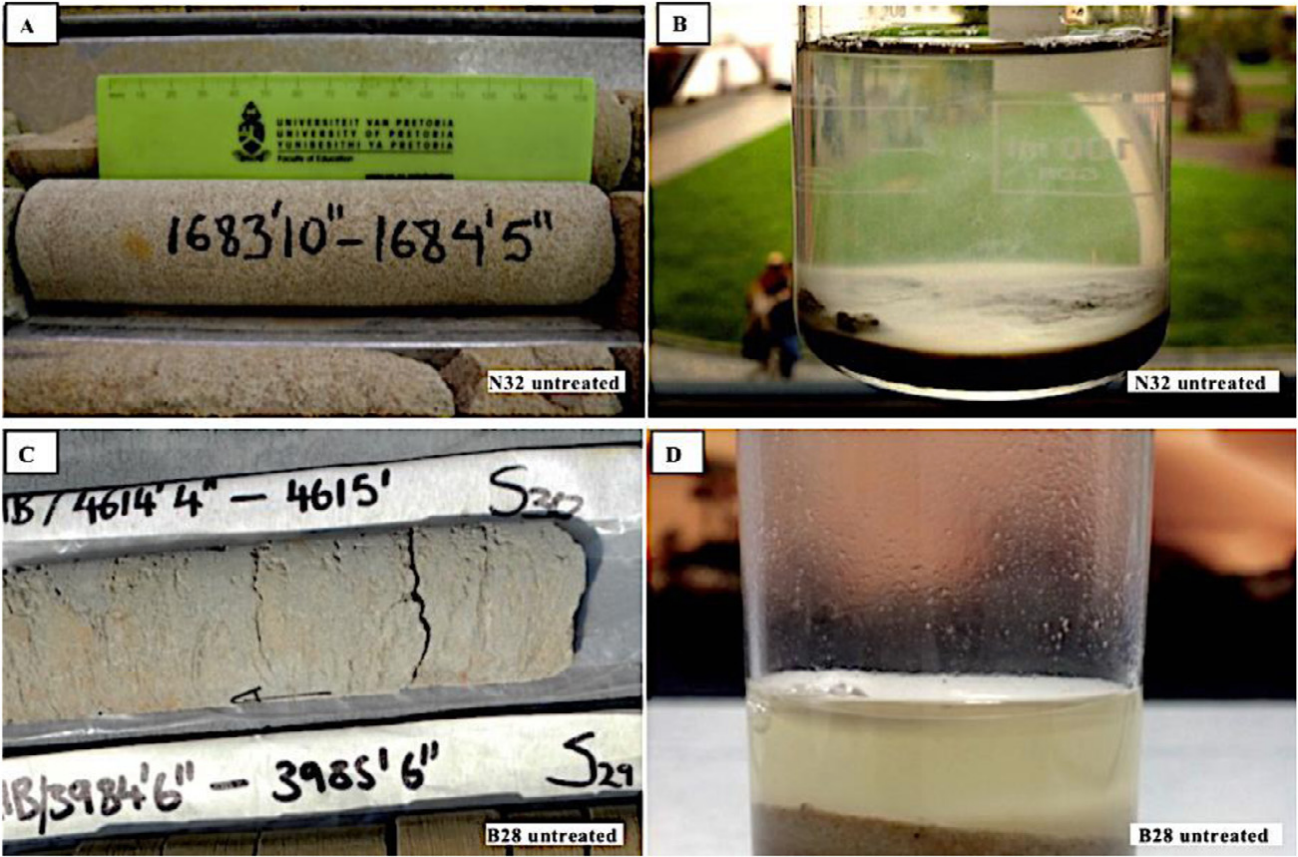
(A) – sample N32 and (C) – sample B28 are calcareous sandstone and have completely disintegrated after being soaked in distilled water for two days (B and D). Untreated means that the samples were not reacted with scCO_2_, whereas treated implies that the samples have been reacted with scCO_2_.

The interaction between CO_2_, pore water and mineral phases involves several different parameters such as the composition of the rocks, fluids and fluids composition, texture of the mineral grains, temperature and pressure [1,4]. The sandstones and siltstones were saturated with pore water for two days under ambient conditions. The data show that calcareous sandstone, e.g. sample N32 (514.96 – 513.71 m) and B28 (1378.00 – 1377.85 m) disintegrated upon contact with pure water within the period of 48 hours without before been treated with CO_2_ (Fig. 5).

The initial sandstone and siltstone lithofacies were placed into autoclaves and reacted with CO_2_ under the temperature of 100 °C and pressure of 100 bars. The sample treatment procedure outlined below was followed for the H_2_O-CO_2_-rock experiments.

1. Examined the initial sediment petrography using optical microscopy.
2. Utilised SEM, EDS and XRD for further analysing of the mineralogy.
3. Saturated the rock samples with distilled water for 48 hours.
4. Placed the samples into autoclaves filled with CO_2_.
5. Placed the autoclaves into the oven under 100 °C and 100 bars.
6. Repeated step 1 through 2 after scCO_2_ treatment.

By repeating the test after step 4, it was possible to quantify and compare changes in the physical and chemical properties before and after treating sandstone and siltstone with scCO_2_. The sample was cut into blocks of approximately 3 × 2 × 1 cm and saturated with distilled water for 2 days. The density, mass, and volume of saturated rock samples were calculated to determine the amount of dry ice needed to simulate the natural geological conditions of a temperature of 100 °C and a pressure of 100 bar. The Kern PRS instrument automatically calculated the density of soaked rock samples by first measuring the mass (in grams) of wet block samples in air and in distilled water at known temperatures and densities. The water density was assumed to be 0.9792 g/cm^3^ at a water temperature of 24.4 °C measured with a thermometer. To calculate the mass of dry ice required to react the rock sample in the autoclave, it was necessary to know the filling volume of each autoclave, which was determined by calibration with water using a measuring pipette. The volume of the autoclave was then used to calculate the mass of CO_2_ required to react with rock samples of known mass and density. Using Peng Robinson's equation of state (Eq. (1)), the volume of 100% pure dry ice as a function of pressure and temperature was determined [5].

Peng-Robinson equation of state

(1) $$P=\frac{RT}{V_{m}-b}-\frac{a}{V_{m}^{2}+2V_{m}b-b^{2}}$$

$$\begin{array}{ccc} a & = & \frac{0.457236a\;R^{2}T_{c}^{2}}{P_{c}},\;b=\frac{0.0777961\;RT_{c}}{P_{c}},\ldots \\ a & = & {\left(1+\left(0.37464+1.54226\omega -0.26992\omega^{2}\right)\left(1-\sqrt{\frac{T}{T_{c}}}\right)\right)}^{2} \end{array}$$

*V_m_* = relative volume, *T* = temperature, *Tc* = critical temperature, *p =* Pressure*, Pc* = critical pressure, ω = acentric factor, *a* = Peng-Robinson constant *a, b* = Peng-Robinson constant *b, R =* molar gas constant

The number of moles (n) of CO_2_ was calculated from the volume of CO_2_ using Eq. (1) and then the moles were used together with the molar mass (Mm) of CO_2_ to determine the mass of CO_2_. The molar mass of CO_2_ is determined from the atomic masses of carbon and oxygen taken from the periodic table of elements: C = 12.011 and O = 15. 999.

(2) $$\text{Mm}\left(CO_{2}\right)=C+2O=12.011+2\left(15.999\right)=44.009\;\text{gram}/\text{mole}$$

(3) $$\text{Then}\;\text{the}\;\text{mass}\;\text{of}\;\text{C}O_{2}\;\text{is}\;\text{given}\;\text{by}\;\text{m}=\text{nMm}$$

*m* is the mass of CO_2_; *n* is the number of moles of CO_2_ determined from Eq. (1) and *Mm* is the molar mass of CO_2_ calculated from Eq. (2).

### 2.8 Petrophysical properties

#### 2.8.1 Optical microscopy

The porosity and permeability of the rock samples investigated were determined by digital analysis of uncovered thin sections. The thin sections were dyed with blue epoxy and were examined with a transmitted light microscope. The exercises were performed before and after processing the sample with scCO_2_. Digital image analysis was performed to visually quantify pore parameters in the range of µm - mm, estimate the volume of open pores, and assess pore connectivity. This method of characterising porosity does not require knowledge of lithology, age and depth of burial, or sediment diagenesis. Digital analysis of coloured uncovered thin section photomicrographs were taken at variable magnification using an optical microscope and provided information on 2D macro porousness, whereas sectioned block samples were utilised to determine 3D micro porousness using SEM.

Quantification of pores and their connectivity by analysing digital images is a very important and well-established technique. Light and electron microscopy images of thin sections were used to separate rocks into pores and solids. In this undertaking, the pore space was impregnated with blue epoxy to produce a binary optical image. By combining an optical microscope and a scanning electron microscope, a method of digital image analysis covering pore sizes from the µm - mm scale was applied and allowed the quantification of pore space with variations in size exceeding three orders of magnitude in the same sample.

The porosity and permeability discrepancies of the various reacted and unreacted sandstone and siltstone samples, the amount of macroporosity, the amount of microporosity in the matrix were then determined for each sample (Table 5). The estimated total porosity is compared to the 7-41% porosity value obtained from the work peformed by Viljoen et al. [2] published in South Africa's CCS Atlas for the middle sandstone analysis [6]. Comparisons were made between porosity measurements and digital image analysis to determine the reliability of digital image analysis technique. Porosity data provide information about pore size distribution and pore shape, and were utilised to explain the distribution of permeability. The data show that the porosity is mainly controlled by the intergranular pore shape in high permeability sandstone layers.

### 2.8.2 Scanning electron microscopy

**Fig. 6:**
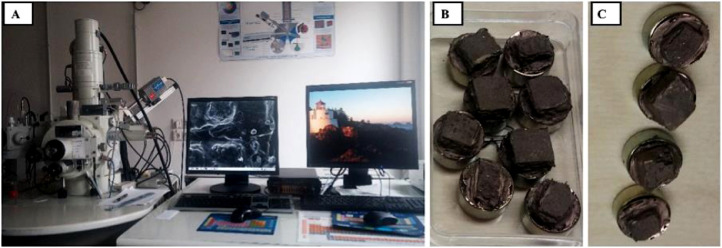
JOEL JSM 6300 scanning electron microscope, institute for geological sciences and geography at the Martin-Luther University (A). Unreacted (B) and reacted (C) core block samples, cut into 1 cm^3^ blocks and mounted on 12 mm by 5 mm aluminium planchets using sticky tabs. They were examined for mineral modifications after the experiments.

Using JEOL JSM-6300 (Fig. 6) at the Martin Luther Universitӓt, Halle-Wittenberg, Germany, 1 cm^3^ unreacted and reacted core samples (Fig. 6B and C) were mounted on JEOL, aluminium 12 mm by 5 mm planchets using sticky tabs and analysed. The JEOL low vacuum capability of the JSM6300 requires the surface of the newly cut samples to be coated with gold, allowing electrons conduction and capturing of high quality micrographs. For imaging, the operating conditions were set to 20 kV and a spot size of 5 µm. Imaging was monitored with a video camera mounted on the scanning electron microscope (SEM). This allowed observation and photographing of particles at various magnifications up to 5000X magnification, although not required for this project. Images were collected with secondary and backscatter detectors, and utilised for digital image analysis for additional information on pore shape, pore size, rock texture, and mineral morphology. The brightness and contrast of SEM photomicrographs provide indicators of compositional changes within the sample and various minerals that appear as contrasting grey shades.

The JEOL JSM6300 SEM is equipped with Energy Dispersive Spectroscopy (EDS) and was used for semi-quantitative elemental analysis. EDS utilised electron beam area of ​​20 kV at a scanning rate of 600 times to determine the local chemical composition of the material. Digital image analysis was performed before and after the water-CO_2_-rock interaction experiments to determine the shape, size, and surface roughness of the mineral particles of specific target such as quartz, calcite, feldspar, and clay. Accurate digital image analysis before and after the water-CO_2_-rock reaction is important for image comparison to detect changes in mineral dissolution, porosity and permeability. Photomicrographs randomly taken on the sample surface and inspected by SEM were then used to compare the reacted and unreacted samples.

Due to autoclave and SEM size restrictions, the exact same samples of unreacted and processed samples could not be used before and after analysis. However, a subset of the samples were used to reassess physical and chemical properties after treating the samples with scCO_2_. The SEM device is connected to a confocal microscope, which allows simultaneous imaging and mineralogy analysis. The confocal configuration reduced the sample volume, from which the morphology, size, and shape of the particles were recorded. The size of the reactive surface of a mineral depends on its roughness parameters. The surface roughness of the recorded grain sample depends on the observation scale, which is very limited to 5 µm in SEM. The digital surface image of the sample provided high depth of focus at µm resolution for quantitative topography data.

### 2.9 Geomechanical investigation

**Fig. 7:**
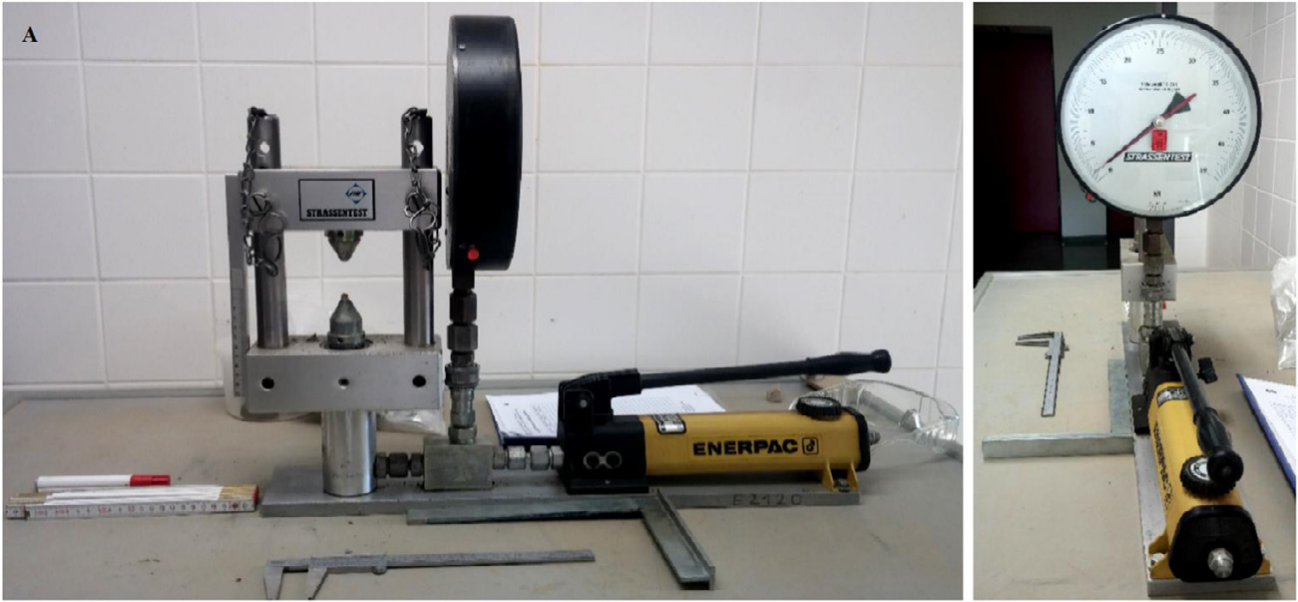
Enerpac point-load tester used to gauge the uniaxial compressive stress of the sandstone and siltstone samples. Enerpac P141 is a single speed, hydraulic hand pump, 327 cm^3^ and 12.7 mm cylinder stroke, operated at the pressure of 700 bar.

Various sandstones and siltstone samples were used in the Enerpac P141 device (Fig. 7), which consisted of a hydraulic hand pump and a plunger. The Enerpac is a single-stage hydraulic hand pump, 327 cm^3^ and 12.7 mm cylinder strokes, operating at 700 bar pressure. In this test, irregularly shaped rock fragments were placed between standard-sized pointed platens and loaded until breakage occurred when compressed. The compressive strength of the rock sample investigated is the force per unit area that the rock sample can withstand during compression [1]. The point load index (Is) was calculated as the ratio of the applied load (P) to the square of the distance (D) between the load points [7].

(4) $$\text{Is}=P/D^{2}$$

Loading point dimensions are standardised (Fig. 7). By using a standardised core diameter of 50 mm, the relationship between point load index and uniaxial compressive strength (UCS) was determined [7,8]. This method provided rapid and reliable laboratory measurements for assessing the strength of unreacted sandstone and siltstone samples that were being investigated for CO_2_ sequestration.

**Fig. 8:**
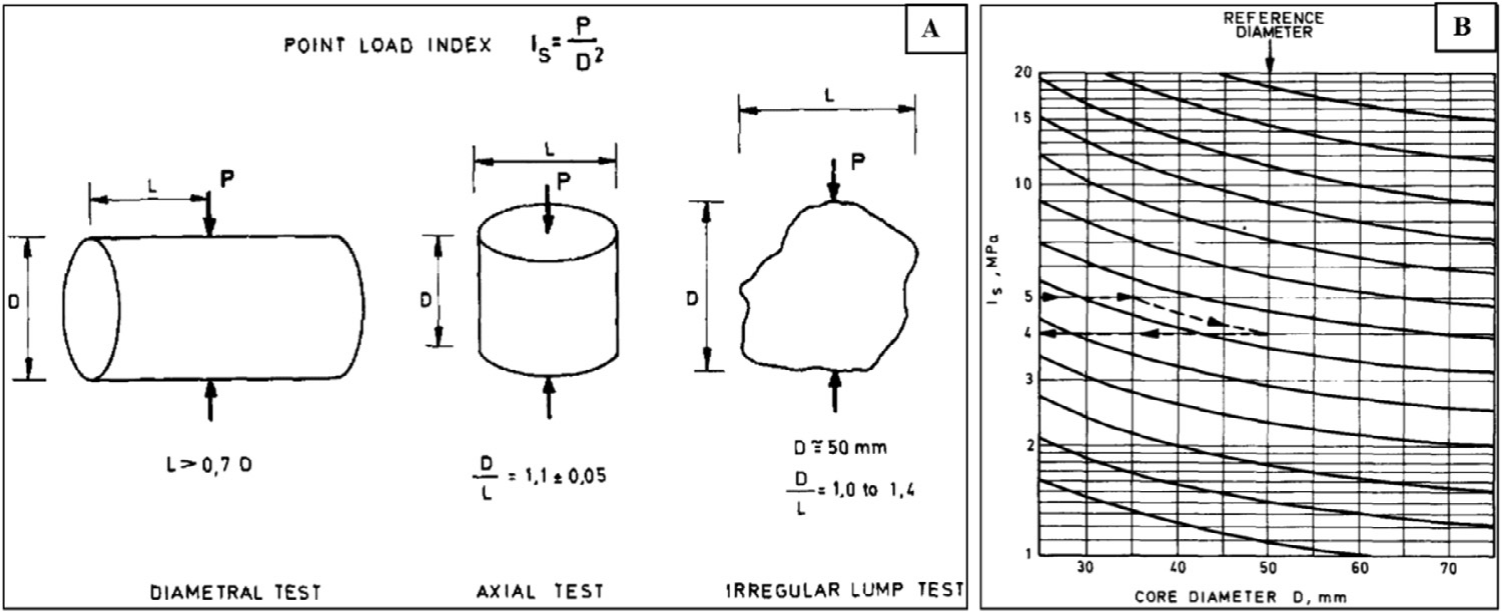
Geometrical conditions for the diametrical, axial and asymmetrical lump testing (A). (B) Illustrates size correlation chart for point-load index suggested by Broch and Franklin [8].

Only broken lumps of rocks were available, tests were performed along two axes, the shortest axis of the mass was parallel to the bedding plane, and the sample diameter was less than 50 mm (Fig. 8A). The longest axis is represented by measurements recorded perpendicular to the sample layer. Broch and Franklin [8] showed that the value of load strength at the diameter point depends on the size of the core. Cores with larger diameters have smaller point load index values. Therefore, it was proposed to make a standard classification by modifying all values ​​to a reference diameter of 50 mm. The correction table used for this purpose is shown in (Fig. 8B).

The size correlation diagram of the point load index (Fig. 8) is proposed by [8], but there are restrictions on this diagram if accurate details are needed. Assuming D = 35 mm, the chart shows Is = 5, and the bend corresponding to the proximity of the chart creates a reference, Is = 4 for the D = 50 mm core. This reference point load index is used for strength classification purposes. Since the point load test was used for strength classification purposes, lithological masses were classified by uniaxial compressive stress in addition to the compressive strength index itself (Table 9).

**Table 9:** Uniaxial compressive stress [7,8].

Description	Uniaxial Compressive Strength (MPa)	Point-load index (MPa)
Very high strength	˃200	˃8
High strength	100 – 200	4 – 8
Medium strength	50 – 100	2 – 4
Low strength	25 – 50	1 – 2
Very low strength	˂ 25	˂ 1

## CRediT Author Statement

**Lowanika Tibane:** Investigation, Writing - Review & Editing, Data Curation, Project administration, Funding acquisition. **Phil Harris**: Resource, Supervision. **Herbert Pöllmann:** Resource, Supervision. **Fillsmith Ndongani:** Sample collection, Investigation. **Brandon Landman:** Data collection, Investigation. **Wlady Altermann:** Conceptualisation, Supervision, Funding acquisition.

## Declaration of Competing Interest

The authors declare that they have no known competing financial interests or personal relationships that could have appeared to influence the work reported in this paper.
